# Overexpression of *OsERF83*, a Vascular Tissue-Specific Transcription Factor Gene, Confers Drought Tolerance in Rice

**DOI:** 10.3390/ijms22147656

**Published:** 2021-07-17

**Authors:** Se Eun Jung, Seung Woon Bang, Sung Hwan Kim, Jun Sung Seo, Ho-Bin Yoon, Youn Shic Kim, Ju-Kon Kim

**Affiliations:** 1 Crop Biotechnology Institute, GreenBio Science and Technology, Seoul National University, Pyeongchang 25354, Korea; seun0204@snu.ac.kr (S.E.J.); seungwoon93@gmail.com (S.W.B.); sunghwan525@gmail.com (S.H.K.); xfiles96@snu.ac.kr (J.S.S.); yoonhobin@snu.ac.kr (H.-B.Y.); younshic@kangwon.ac.kr (Y.S.K.); 2 Agriculture and Life Sciences Research Institute, Kangwon National University, Chuncheon 24341, Korea

**Keywords:** drought tolerance, plant growth, *Oryza sativa*, *OsERF83*, ERF transcription factor, vascular tissue, CRISPR/rCas9

## Abstract

Abiotic stresses severely affect plant growth and productivity. To cope with abiotic stresses, plants have evolved tolerance mechanisms that are tightly regulated by reprogramming transcription factors (TFs). APETALA2/ethylene-responsive factor (AP2/ERF) transcription factors are known to play an important role in various abiotic stresses. However, our understanding of the molecular mechanisms remains incomplete. In this study, we identified the role of *OsERF83*, a member of the AP2/ERF transcription factor family, in response to drought stress. OsERF83 is a transcription factor localized to the nucleus and induced in response to various abiotic stresses, such as drought and abscisic acid (ABA). Overexpression of *OsERF83* in transgenic plants (*OsERF83^OX^*) significantly increased drought tolerance, with higher photochemical efficiency in rice. *OsERF83^OX^* was also associated with growth retardation, with reduced grain yields under normal growth conditions. *OsERF83* is predominantly expressed in the vascular tissue of all organs. Transcriptome analysis revealed that *OsERF83* regulates drought response genes, which are related to the transporter (*OsNPF8.10, OsNPF8.17, OsLH1),* lignin biosynthesis (*OsLAC17, OsLAC10, CAD8D)*, terpenoid synthesis (*OsTPS33, OsTPS14, OsTPS3)*, cytochrome P450 family (*Oscyp71Z4, CYP76M10*), and abiotic stress-related genes (*OsSAP, OsLEA14, PCC13-62). OsERF83* also up-regulates biotic stress-associated genes, including *PATHOGENESIS-RELATED PROTEIN* (*PR*), *WALL-ASSOCIATED KINASE* (*WAK*), *CELLULOSE SYNTHASE-LIKE PROTEIN E1* (*CslE1*), and *LYSM RECEPTOR-LIKE KINASE* (*RLK*) genes. Our results provide new insight into the multiple roles of *OsERF83* in the cross-talk between abiotic and biotic stress signaling pathways.

## 1. Introduction

Abiotic stresses, including drought, cold, salinity, and nutrient stress, adversely affect the cellular homeostasis of plants and ultimately impair their growth and productivity. Among these abiotic stresses, extreme heat and water deficits frequently damage plants [1]. As extreme weather disasters associated with climate change have become steadily more common, crop losses have also increased over the past several decades [2,3]. Rice (*Oryza sativa.* L.) productivity is severely affected by drought because rice typically requires more water than other crops [4]. It is, therefore, essential to develop rice with enhanced tolerance to drought and heat stress.

To avoid drought stress, plants have evolved a series of sophisticated strategies. Plants respond to drought stresses through stress-specific signaling pathways, leading to morphological, physiological, and biochemical changes. Under drought stresses, plants first perceive external signals through sensors, and then various transcription factors are induced by the transduction of signals [5,6]. These drought-responsive transcription factors, such as members of the AP2/ERF, MYB, bZIP, and NAC families, regulate drought-inducible genes, and consequently, plants show a tolerance to abiotic stresses [5].

The *OsERF83* gene belongs to the APETALA2/ethylene-responsive factor (AP2/ERF) family, which is only present in the plant kingdom [7]. The AP2/ERF superfamily commonly possesses a highly conserved AP2 DNA-binding domain, and can be classified into four subfamilies: APETALA2 (AP2), related to abscisic acid insensitive 3/viviparous 1 (RAV), ethylene-responsive factor (ERF), and dehydration-responsive element binding protein (DREB) [8]. In the AP2/ERF superfamily, the ERF family has a single DNA-binding AP2 domain and is classified into ten subgroups (Ⅰ–X) based on putative functional motifs [7]. Many TFs belonging to the ERF family have been reported that specifically bind to the GCC box (AGCCGCC), an ethylene-responsive element (ERE) [7], to play a vital role in plant development, abiotic and biotic stresses, and hormonal signal transduction [9,10,11,12,13,14]. Recently, *OsERF48* and *OsERF71* were shown to be involved in drought tolerance in rice by enhancing root growth [15] and altering the root structure [16], respectively. Additionally, overexpression of *OsEREBP1* confers tolerance to both biotic and abiotic stresses [17]. In Arabidopsis, *AtERF019* has been reported to play an important role in plant growth and drought tolerance by delaying plant growth and senescence [18]. However, despite increasing evidence that *OsERF* genes enhance drought tolerance, the molecular mechanisms have not yet been entirely elucidated.

TFs belonging to group Ⅸ are associated with defense against various pathogens [7]. For example, overexpression of *AtERF1* and *ORA59* enhances resistance to necrotrophic pathogens in Arabidopsis. *AtERF1* is a regulator of ethylene responses after pathogen attack, and *ORA59* is an essential integrator of jasmonic acid (JA) [19,20]. In rice, *OsERF92* negatively regulates tolerance to *Magnaporthe oryzae* and salt stress, which is integrated into the cross-talk between biotic and abiotic stress signaling networks [21]. *OsERF83*, which belongs to Group Ⅸ, is induced by drought, high salinity, low temperature, and ABA treatments [9]. A previous study demonstrated that *OsERF83* is a transcription factor that positively regulates resistance to *Magnaporthe oryzae* and is induced by multiple phytohormone treatments, such as methyl jasmonate, ethephon, and salicylic acid. Furthermore, several *PATHOGENESIS-RELATED* (*PR*) protein genes, including *PR1*, *PR2*, *PR3*, *PR5*, and *PR10*, are up-regulated in *OsERF83* overexpression (*OsERF83^OX^*) transgenic rice [22]. These results prompted us to study the function of *OsERF83* in biotic and abiotic stresses. In this study, we found that *OsERF83* confers drought tolerance and positively regulates abiotic and biotic stress-associated genes.

## 2. Results

### 2.1. OsERF83 Is a Drought-Inducible Transcription Factor

**Figure 1:**
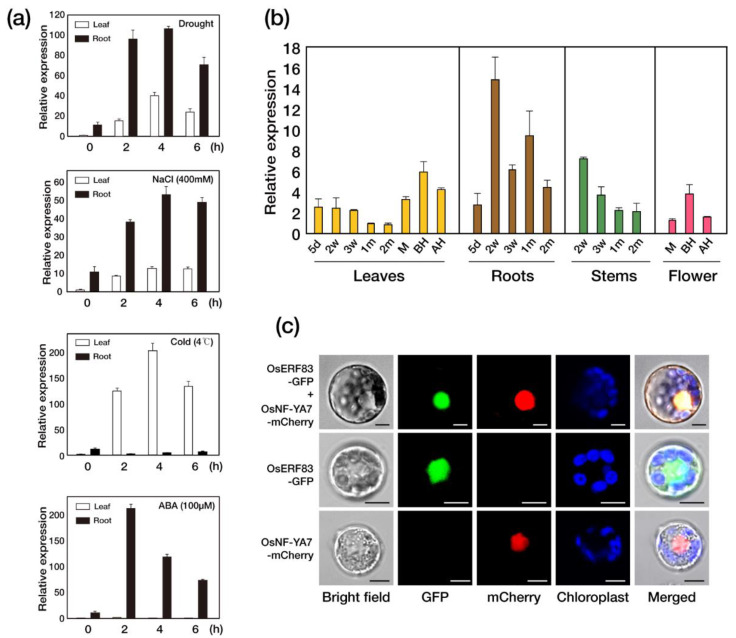
Expression pattern of *OsERF83* and subcellular localization of OsERF83 in rice protoplasts. (**a**) The relative expression level of *OsERF83* by quantitative RT-PCR in the roots and shoots of 2-week-old seedlings treated with air-drying (drought), 400 mM NaCl (high salinity), 4 °C (cold), and 100 μM abscisic acid (ABA). *OsUbi* (*Ubiquitin1*; Os06g0681400) expression was used as an internal control for normalization. Error bars indicate the standard deviation based on three technical replicates. (h: hour) (**b**) The expression level of *OsERF83* in various tissues and at different growth stages. (d, day; w, week; m, month; M, meiosis; BH, before heading; AH, after heading). *OsUbi1* expression was used as an internal control for normalization. Error bars indicate the standard deviation based on three technical replicates. (**c**) Subcellular localization of the OsERF83 protein in rice protoplasts. *35S::OsERF83-GFP* and *35S::OsNF-YA7-mCherry* as a control vector were transiently expressed in rice leaf protoplasts. Scale bar, 10 µm.

In our previous rice 3′-tiling microarray analysis, *OsERF83* was induced by drought treatments [9]. Based on the results, we investigated the function of *OsERF83* in response to drought. We analyzed the expression patterns using quantitative real-time PCR (qRT-PCR) (Figure 1a). We treated 2-week-old rice seedlings with various stresses, including drought, high salinity, low temperature, and ABA. The transcript level of *OsERF83* was significantly induced in both leaves and roots under drought and high salinity conditions, and the degree of induction was higher in the roots than in the leaves. In contrast, the low-temperature (4 °C) and ABA (100 μM) treatments enhanced the transcript levels of *OsERF83*, specifically in the leaves and roots, respectively. Under drought treatments, the transcript levels of *OsERF83* were increased in both the roots and leaves (Figure 1a). The regulation of *OsERF83* expression by various stresses shows that it might have multiple roles in response to environmental stresses.

To investigate the spatiotemporal expression patterns of *OsERF83*, we conducted qRT-PCR with various tissues of rice. The results revealed that *OsERF83* was expressed at all developmental stages, and at particularly high levels in the roots (Figure 1b).

One study reported that *OsERF83* was localized to the nucleus in onion epidermal cells [22]. To confirm the subcellular localization of *OsERF83* in rice, we transformed the full-length *OsERF83,* fused to green fluorescent protein (GFP), into rice protoplasts (Figure S1a). We also used OsNF-YA7-mCherry as a positive control for nucleus localization [23]. Both constructs were transiently co-expressed in rice protoplasts, and the signals of both green fluorescence and mCherry fluorescence were observed in the nucleus of the rice protoplasts (Figure 1c). This indicated that the OsERF83 was localized at the nucleus. Collectively, OsERF83 is a transcription factor responsive to multiple stresses.

### 2.2. Overexpression of OsERF83 in Rice Confers Drought Tolerance at the Vegetative Stage

To investigate the biological role(s) of *OsERF83* in rice development and the drought-stress response, we generated over 30 independent *OsERF83* overexpression (*OsERF83^OX^*) transgenic rice plants using the *GOS2* (rice eukaryotic translation initiation factor 1-like gene) promoter (*GOS2::OsERF83*), which drives expression in the whole plant body [24] (Figure S1b). Initially, we selected single-copy homozygous lines of *OsERF83^OX^* using TaqMan PCR, and then somaclonal variations were eliminated by paddy field selection for three generations. Finally, we chose three homozygous elite lines of *OsERF83^OX^* (#1, 2, 11) for further study. The transcript levels of *OsERF83* were greatly enhanced in both the leaves and roots of all *OsERF83^OX^* (#1, 2, 11) plants, compared to non-transgenic (NT) plants. We checked the expression of each transgenic sister line and confirmed their overexpression (Figure S3).

**Figure 2:**
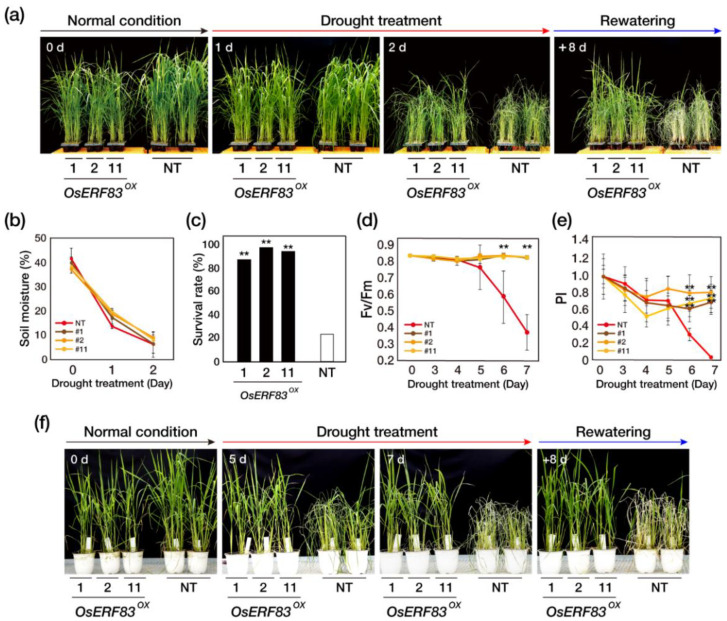
Overexpression of *OsERF83* in rice confers drought tolerance. (**a**) Drought tolerance phenotypes of *OsERF83* overexpression (*OsERF83^OX^*) transgenic plants (lines 1, 2, 11). The 5-week-old plants (0 d) were exposed to drought by withholding water for 2 days and rewatering for 8 days. (**b**) Measurement of soil moisture content (%). Error bars represent the means ± SD (*n* = 10). (**c**) The survival rate of the transgenic plants, scored 8 days after rewatering. (**d**,**e**) Determination of the photosynthetic viability of transgenic and NT plants under drought conditions. At the indicated time point after exposure to drought stresses, the chlorophyll fluorescence (Fv/Fm) (**d**) and performance index (PI) (**e**) of overexpression plants and NT plants were measured. The data represent the mean ± SD (*n* = 10 points per independent line of each genotype). (**f**) Drought tolerance phenotypes of *OsERF83^OX^* transgenic plants (lines 1, 2, 11) in big pots. The 6-week-old plants (0 d) were exposed to drought by withholding water for 7 days and rewatering for 8 days. Asterisks (**) indicate statistically significant differences compared with non-transgenic (NT) plants according to a Student’s *t*-test ** *p* < 0.001).

We next tested the performance of *OsERF83^OX^* under drought conditions at the vegetative stage. *OsERF83^OX^* and NT plants were grown in a greenhouse for 5 weeks and then exposed to drought conditions for 2 days by withholding water. After 1 day of drought treatment, the NT plants started to display slightly rolled leaves. After 2 days of drought treatment, drought-induced visual symptoms, such as leaf rolling, wilting, and chlorosis, appeared more severely in NT plants than *OsERF83^OX^* (#1, 2, 11) plants (Figure 2a). Soil moisture consistently decreased during the drought treatment, indicating the drought stress was uniformly applied to both the NT and *OsERF83^OX^* (Figure 2b) plants. After 8 days of rewatering, most of the *OsERF83^OX^* plants had recovered and showed an 83 to 95% survival rate, while NT plants showed an approximately 20% survival rate (Figure 2c). We repeated the testing three times through T3–T4 and obtained the same results (Figure S4).

To further confirm the tolerance of *OsERF83^OX^* plants to drought stress, we measured the activity of photosynthetic machinery damaged by drought stress using the JIP test. The JIP test showed the *Fv/Fm* (Fv: variable fluorescence; Fm: maximum fluorescence) value for the photochemical efficiency of photosystem (PS) II and the PI_total_ (performance index) value for the photochemical efficiency of PS I and PS II [25]. *OsERF83^OX^* and NT plants were exposed to drought conditions for 7 days after transplanting into big pots, by withholding water for 7 days (Figure 2f). The *Fv/Fm* values of the NT plants started to decrease at 5 days after drought treatment, whereas the *Fv/Fm* values of *OsERF83^OX^* plants did not decrease (Figure 2d). Additionally, the PI_total_ values sharply decreased in NT plants at 5 days after drought treatment, whereas the PI_total_ values of *OsERF83^OX^* were maintained during the drought treatment (Figure 2e). Consistent with the photochemical efficiency, after 5 days of drought treatment, the NT plants started to display drought-induced visual symptoms (Figure 2f). These results indicated that *OsERF83* overexpression in rice plants protects PS I and PS II from drought stress, thereby enhancing drought tolerance; in this way, *OsERF83* positively regulates drought tolerance. We concluded that overexpression of *OsERF83* enhanced drought tolerance at the vegetative stage.

### 2.3. Overexpression of OsERF83 in Rice Leads to Growth Retardation and Affects Grain Yield under Normal Conditions

**Figure 3:**
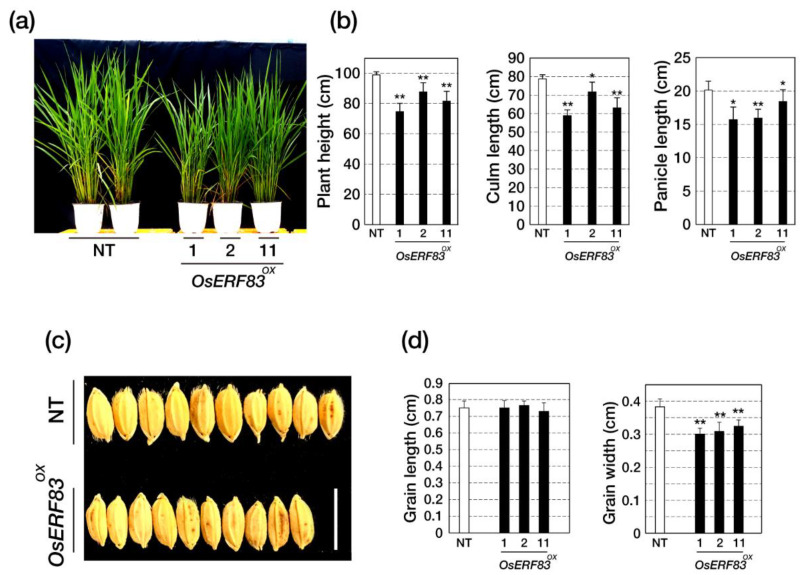
Morphological analysis of *OsERF83^OX^* transgenic plants. (**a**) The whole plant height of 2-month-old *OsERF83^OX^* and NT plants grown in paddy fields. (**b**) The plant height was measured after the heading stage of the rice. A total of 18 plants for each line were measured. Data represent the mean value ± SD (*n* = 10). (**c**,**d**) The grain size of *OsERF83^OX^* line and NT plants. A total of 25 grains from each line were measured. Bar = 1 cm. Two asterisks (**) indicate statistically significant differences at *p* < 0.01, and one asterisk (*) indicates significant differences at *p* < 0.05 compared with NT based on the Student’s *t*-test. Data are shown as the mean ± SD (*n* = 25).

The *OsERF83^OX^* plants showed smaller plant height, including culm length and panicle length at all stages (Figure 3a,b). At the maturity stage, the average plant height of NT, *OsERF83^OX^* #1, *OsERF83^OX^* #2, and *OsERF83^OX^* #11 was 98.86 cm (100%), 75.50 cm (76.4%), 87.63 cm (87.6%), and 81.50 cm (81.5%), respectively (Figure 3b, Table S1). The yield component values of the *OsERF83^OX^* plants, such as the number of spikelets per panicle (NSP), the number of total spikelets (NTS), the number of filled grains (NFG), the grain-filling rate (FR), and total grain weight (TGW), were reduced compared to the NT plants (Figure S5, Table S1). Moreover, the *OsERF83^OX^* plants showed shorter grain width than the NT plants (Figure 3c,d). Collectively, these results indicate that overexpression of *OsERF83* affects plant growth and grain yield negatively in rice.

### 2.4. CRISPR/Cas9-Mediated Loss-of-Function Study Analysis of OsERF83

**Figure 4:**
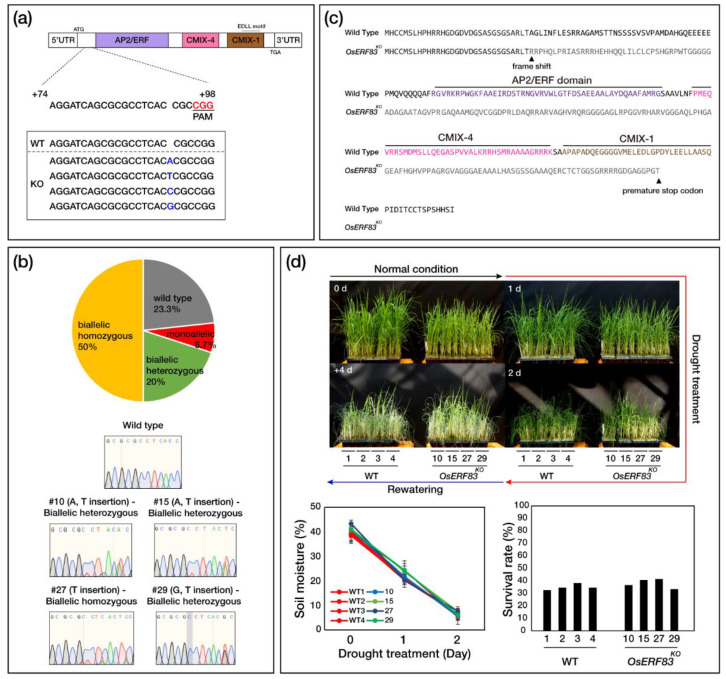
Construction of knock-out (*OsERF83^KO^*) mutants and their phenotypes. (**a**) Schematic illustration of the target sites in the OsERF83 genomic sequence. The protospacer adjacent motif (PAM) sequence is marked in red. Mutation sequences are marked in blue. (**b**) Mutation pattern on *OsERF83^KO^* #10, #15, #27, and #29 plants. (**c**) Amino acid sequence alignment of OsERF83KO plants revealed a frameshift and premature stop codon. (**d**) Phenotypes of *OsERF83^KO^* (#10, #15, #27, #29) and WT plants under drought stress. All plants were grown in soil for 5 weeks under well-watered conditions, exposed to drought stress for 2 days, and then rewatered for 4 days, in the greenhouse. Measurement of the soil moisture contents (%). The data represent the mean value ± SD of five measurements performed at different locations in the soil. The survival rate of transgenic plants was scored 4 days after rewatering.

For the loss-of-function study analysis, knock-out (*OsERF83^KO^*) mutants were generated using a clustered regularly interspaced short palindromic repeats/recombinant codon-optimized Cas9 (CRISPR/Cas9) genome editing system (Figure S1c) [26], with which various types of induced mutations were found. The single-guide RNA (sgRNA) target sites were designed at the upstream region of the AP2/ERF domain for generating mutants edited in *OsERF83* specific regions (Figure 4a). Four types of mutants containing 1 bp insertions were generated at the same upstream position of the PAM sequence (Figure 4a,b). Sequencing analysis revealed mutation patterns in transgenic plants, including frameshifts and premature stop codons (Figure 4c). Among 30 independent transgenic plants, 23.3% were null, and 20% were biallelic heterozygous mutant plants. A further 50% were biallelic homozygous, and we selected four independent *OsERF83^KO^* mutants for further study (Figure 4b).

We expected that the phenotype of *OsERF83^KO^* mutants would show more sensitivity to drought stress compared with NT plants. However, *OsERF83^KO^* mutants showed similar phenotypes under drought conditions (Figure 4d). We speculated that the redundancy of other AP2/ERF transcription factor family genes caused such no significant phenotypes to occur in *OsERF83^KO^* compared to NT plants.

### 2.5. OsERF83 Is Expressed Predominantly in the Vascular Tissue

**Figure 5:**
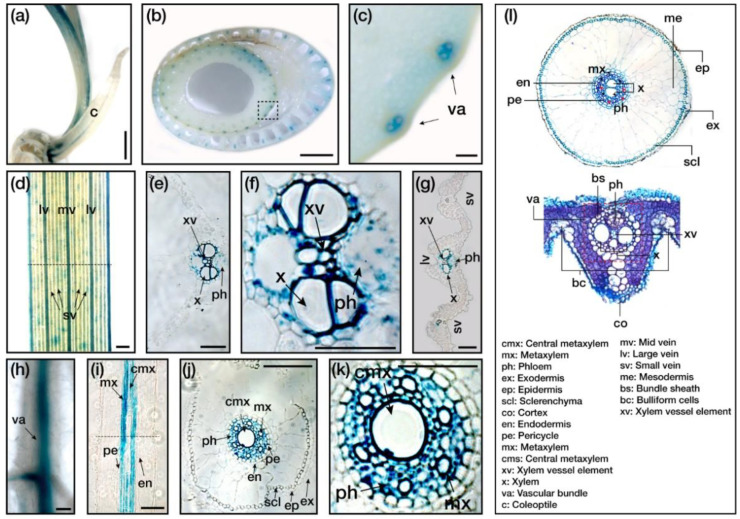
Histochemical analysis of *OsERF83*. (**a**) The *OsERF83* expression in different tissues of the *OsERF83::GUS* transgenic rice plants by GUS staining analysis, in the coleoptile of 5-day-old seedlings (**a**), stem cross-sections of 2-month-old plants (**b**,**c**), leaves of 2-week-old plants (**d**), leaf cross-sections of 2-week-old plants (**e**,**g**), the root of a 5-day-old seedling (**h**), longitudinal root section of 2-week-old plants (**I**), and root cross-sections of 2-week-old plants (**j**,**k**). Scale bars represent 1 mm in (**a**,**b**), 100 µm in (**c**,**d**) and (**h**), and 50 µm in (**e**–**k**). (**i**) Transverse section images represent structures of the roots and leaves.

As shown in Figure 1b, *OsERF83* was expressed ubiquitously in all the examined tissues, with high expression levels in roots. To further explore the function of *OsERF83* in drought tolerance, we investigated the tissue expression pattern of *OsERF83* using histochemical GUS staining with Paraplast section. We generated transgenic rice plants with a β-glucuronidase (*GUS*) reporter gene, driven by the native *OsERF83* promoter (*OsERF83::GUS*) (Figure S1d). We used a coleoptile, stem, leaves, and roots of plants, as exhibited in Figure 5a–k. Interestingly, the strong GUS signals were consistently detected in vascular tissues of the coleoptile (Figure 5a), stem (Figure 5b,c), leaves (Figure 5d–g), and roots (Figure 5h–k), as shown in the longitudinal section of the roots (Figure 5i), despite the difference in developmental stage and organs. These data indicated that *OsERF83* was a constitutively expressed gene and was involved in the vascular tissues.

To investigate possible morphological changes in the vascular tissue caused by *OsERF83* expression, we observed the vascular tissue in the roots, stems, and leaves in *OsERF83^OX^*, *OsERF83^KO^*, and NT plants. However, there were no significant differences in morphology among NT, *OsERF83* overexpressing, and knock-out plants (Figure S6).

### 2.6. Identification of Genes Involved in the OsERF83-Mediated Drought Tolerance Pathway

**Figure 6:**
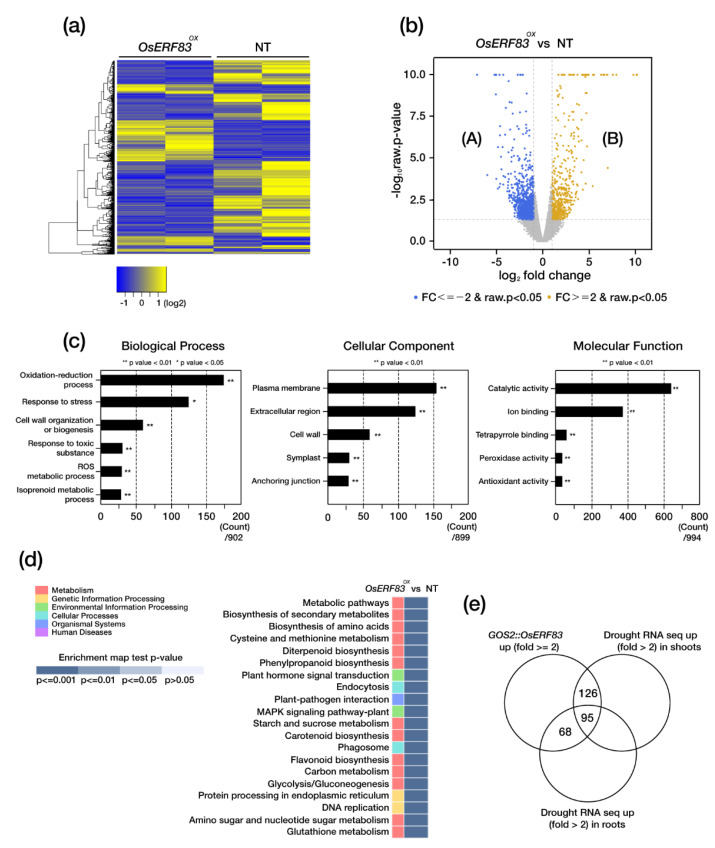
RNA-seq analysis using leaves of *OsERF83-MYC^OX^* plants. (**a**) Heat map analysis of differentially expressed genes (DEGs) between *OsERF83-MYC^OX^* and NT plants. (**b**) Venn diagram of the numbers of up- and down-regulated DEGs in *OsERF83-MYC^OX^* plants compared to NT plants. (**c**) Gene ontology (GO) enrichment analysis of DEGs between *OsERF83-MYC^OX^* and NT plants. The most enriched GO term is shown in ‘biological process’, ‘molecular function’, and ‘cellular component’. Asterisks indicate significant enriched GO terms (** *p* < 0.01, * *p* < 0.05). (**d**) KEGG pathway enrichment analysis of DEGs. (**e**) Venn diagram of up-regulated genes amongst the DEGs identified in *OsERF83-MYC^OX^* and DEGs identified in drought-treated NT shoots and roots from public data (TENOR: http://tenor.dna.affrc.go.jp/, accessed on 30 November 2020).

To perform further functional analysis of *OsERF83*, we generated *OsERF83*-6myc overexpressing transgenic (*OsERF83-MYC^OX^*) plants (Figure S1e). We confirmed protein expression levels of *OsERF83* in *OsERF83*-*MYC^OX^* using Western blot analysis (Figure S7a,f) and transcript levels using qRT-PCR (Figure S7b). Both levels were higher in *OsERF83-MYC^OX^* than in the NT plants. *OsERF83-MYC^OX^* plants also showed drought tolerance in the vegetative stage (Figure S7c–e). To identify downstream genes regulated by *OsERF83*, we performed an RNA-sequencing (RNA-seq) analysis using a 3-week-old *OsERF83-MYC^OX^*. We used whole seedlings of *OsERF83-MYC^OX^* and NT, with two independent biological replicates, and calculated the reads per transcript kilobase per million fragments mapped reads (FPKM) values for each sample with count data. The heatmap indicated that the biological replicates showed a similar expression pattern (Figure 6a). A total of 33,221 differentially expressed genes (DEGs) were identified, of which 540 were up-regulated (B) and 1109 were down-regulated (A) in *OsERF83* overexpressing plants (Figure 6b).

To identify the putative functions of these DEGs, we conducted gene ontology analysis using gProfiler (https://biit.cs.ut.ee/gprofiler/orth). The DEGs were categorized into three categories: biological process, molecular function, and cellular component. As a result, the significantly enriched GO terms were mainly associated with the oxidation–reduction process and stress response under the biological process category, plasma membrane and the extracellular region under the cellular component category, and catalytic activity and ion binding under the molecular function category (Figure 6c). In addition, KEGG pathway analysis showed that DEGs were related to ‘diterpenoid biosynthesis’, ‘phenylpropanoid biosynthesis’, and ‘plant hormone signal transduction’ (Figure 6d). These results are consistent with our proposed role of *OsERF83* in the regulation of drought stress tolerance.

**Figure 7:**
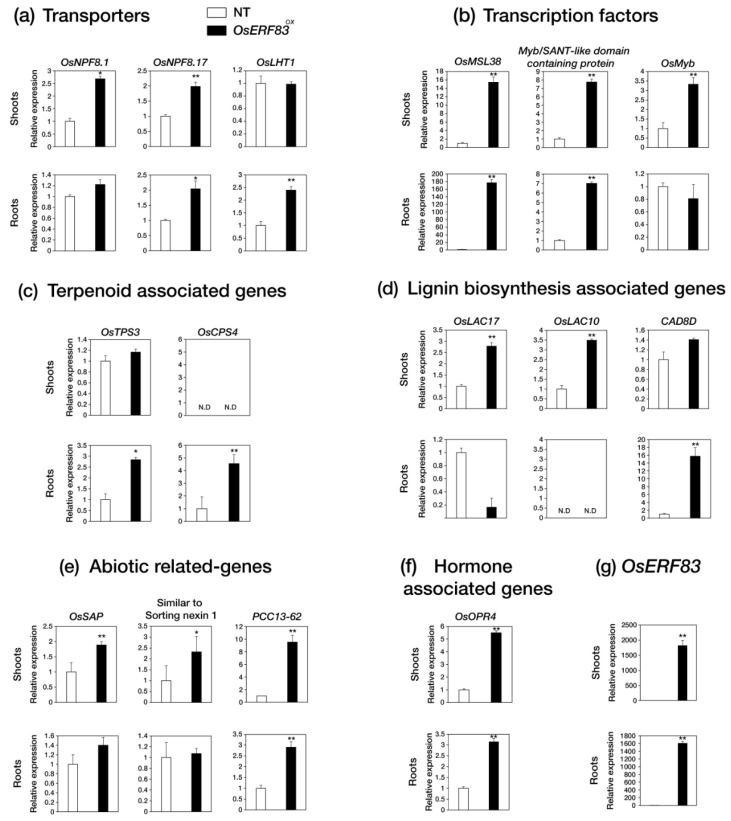
Expression analysis of up-regulated genes in both *OsERF83* overexpression myc-tagged transgenic (*OsERF83-MYC^OX^*) plants and drought-treated samples. (**a**) Relative expression levels of transporter genes (**b**), transcription factors (**c**), terpenoid-associated genes (**d**), lignin biosynthesis-associated genes, (**e**), abiotic stress-related genes. (**f**) and hormone associated genes, (**g**) qRT-PCR analysis of *OsERF83* expression in 3-week-old *OsERF83^OX^* (line 1). The white bar is the expression value in NT plants, and the black bar is the expression value in *OsERF83^OX^* plants. *OsUbi* (*Ubiquitin1*; Os06g0681400) expression was used as an internal control for normalization. Error bars indicated the standard deviation based on three technical replicates. Asterisks indicate significant enriched GO terms (* *p* < 0.01, *p* < 0.05).

Then, we identified that 289 drought-inducible genes were up-regulated in the *OsERF83-MYC^OX^* plants by filtering with a public database containing drought-treated RNA-seq data in shoots and roots (Figure 6e) [27]. These drought-inducible genes were classified into several groups: transporters, transcription factors, abiotic stress-related genes, the cytochrome P450 family, terpenoid-associated genes, disease resistance-related genes, hormone-associated genes, lignin biosynthesis-associated genes, and F-box proteins (Table 1), as well as others (Table S2). Some of the genes in each group were analyzed using qRT-PCR analysis for validation (Figure 7). The results confirmed that *OsERF83-MYC^OX^* induces a class of transporter genes (*OsNPF8.10*, *OsNPF8.17*, and *OsLHT1*) (Figure 7a), MYB or Myb/SANT transcription factors (Figure 7b), terpenoid-associated genes (*OsTPS3* and *OsCPS4*) (Figure 7c), and lignin biosynthesis-associated genes (*OsLAC17*, *OsLAC10*, and *CAD8D*) (Figure 7d). We drew the co-expression matrix using up-regulated genes in *OsERF83-MYC^OX^* using RiceFREND to identify coregulatory networks with a high co-expression frequency (mutual rank (MR): ~20) (Figure S8) [28]. There were many drought-inducible genes in the co-expression matrix (Figure S8).

A previous study reported that *OsERF83* interacts with the GCC box [22]. Therefore, we analyzed the presence of the GCC box within 3 kb of the 5′ upstream region of up-regulated drought-inducible genes in *OsERF83-MYC^OX^* (Table 1, Table S2). We found that most of them have the GCC box, which indicates that *OsERF83* might directly regulate them.

## 3. Discussion

A number of studies have shown that APETALA2/ethylene-responsive factor (AP2/ERF) TFs are involved in the integration of signaling pathways in abiotic and biotic stress responses [10,11]. AP2/ERF TFs are classified into several subfamilies, including the ERF family [8]. *OsERF83* is a member of group IXc, a subgroup of the ERF family reported to play an important role in abiotic stress [19,20,21]. ERF TFs have been reported that regulate stress-inducible genes by binding *cis*-acting elements, such as GCC box (AGCCGCC) in the promoter [29]. Previous studies have demonstrated that OsERF83 is a positive transcriptional regulator and interacts with the GCC box [22], and we analyzed the cis-element GCC box in the promoter of DEGs in our search for putative direct targets (Table 1 and Table S2).

The expression of *OsERF83* was induced by ABA treatment (Figure 1a). In addition, *OsERF83* was also reported to be induced by exogenous SA, JA, and ET [22]. These results indicated that *OsERF83* is involved in multiple phytohormone signaling pathways during environmental stresses. It is well known that there are complex interactions between various hormones and stress signaling pathways in plants [6]. A single gene may play roles in various signaling pathways at the same time [21]. Therefore, our study suggested that *OsERF83* is a positive regulator in plant responses to abiotic and biotic stresses through various hormone signaling pathways.

*OsERF83^OX^* plants showed growth retardation and low grain yields (Figure 3, Figure S5, Table S1). Generally, plants exhibiting stress tolerance have been reported to show growth retardation [30,31] because of hypersensitivity in responding to stresses, directing resources to protection from stresses rather than growing or yielding, which is known as a trade-off. Thus, genes improving stress tolerance induce growth retardation under normal growth conditions when they are constitutively overexpressed. In this study, we used the GOS2 promoter for constitutive overexpression (Figure S1b). However, if we used the stress-inducible promoter instead of the constitutive promoter, *OsERF83* could be a good candidate for crop biotechnology.

The essential functions of vascular tissue are transporting water and various nutrients and supporting structures [32]. Consistent with this, there have been reports that vascular tissue development is involved in drought tolerance [33,34]. *OsERF83* is predominantly expressed in vascular tissue (Figure 5). However, there were no significant differences in phenotypes of vascular tissue in this study (Figure S6). On the contrary, several transcriptome studies have shown that *OsERF83* is induced under iron [35,36] and nitrate deficiency [37]. Moreover, our study revealed that various transporters were up-regulated in *OsERF83-MYC^OX^* (Table 1). Therefore, we speculated that *OsERF83* might also be involved in iron and nitrate absorption.

Previously, it was reported that *OsERF83* positively regulates disease resistance, and the constitutive overexpression of *OsERF83* in rice enhanced resistance to *Magnaporthe oryzae* [22]. However, no further report on the function of *OsERF83* in abiotic stress tolerance has been reported so far. In the present study, we analyzed the function of *OsERF83* as a regulator of responses to drought stress. *OsERF83* was strongly induced not only by drought stress, but also other abiotic stresses (Figure 1a). The *OsERF83* promoter also contains several stress-related *cis*-elements, including ABREs, DREs regulated by ABA and DREB (Figure S9) [38]. These results support *OsERF83* potentially playing an important role in abiotic stresses. *OsERF83^OX^* plants showed drought tolerance under drought treatment in the vegetative stage (Figure 2), which suggested the positive role of *OsERF83* in drought tolerance. While *OsERF83^KO^* mutants showed similar phenotypes compared with non-transgenic (NT) plants under drought treatment at the vegetative stage (Figure 4), this could be explained by functional redundancy among a large number of the AP2/ERF TFs [7,12].

**Figure 8:**
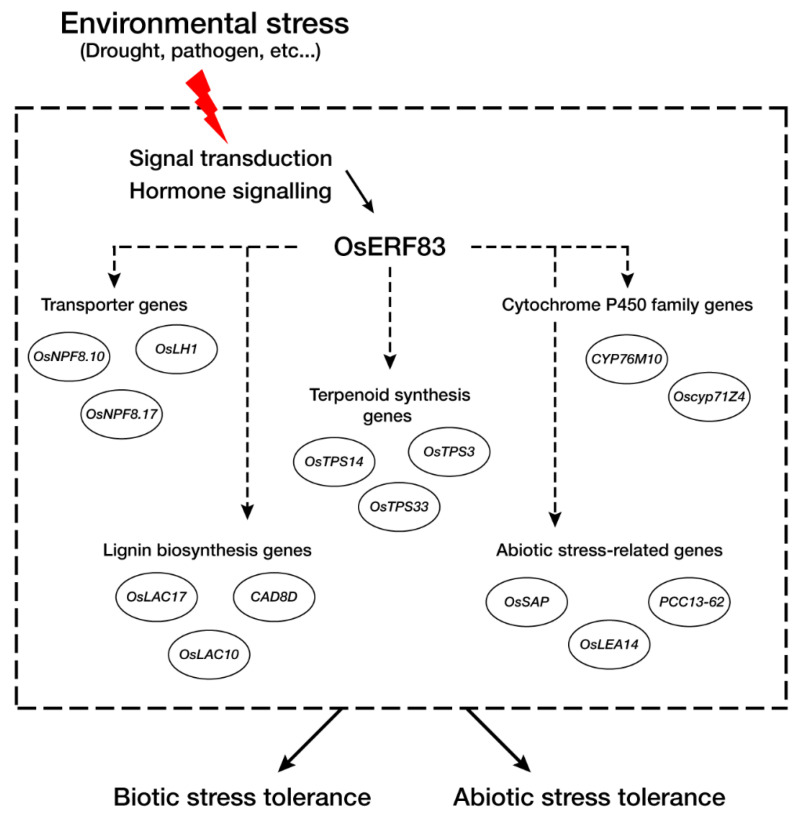
Schematic representation of *OsERF83*-mediated stress tolerance. The rice recognizes the stress signal through a plant hormone signaling pathway, and *OsERF83* is up-regulated in response to stresses. Transporter genes (*OsNPF.10, OsLH1, OsNPF8.17)*, terpenoid synthesis genes (*OsTPS14, OsTPS33, OsTPS3*), cytochrome P450 genes (*Oscyp71Z4, CYP76M10)*, lignin biosynthesis genes (*OsLAC17, OsLAC10, CAD8D*), and abiotic stress-related genes (*OsSAP, OsLEA14, PCC13-62)* were induced by *OsERF83*.

RNA-seq analysis identified a large number of transporter genes that were up-regulated in *OsERF83* overexpression myc-tagged transgenic (*OsERF83-MYC^OX^*) plants, consistent with the vascular-specific expression of *OsERF83*. In a recent report, *NRT1.2*/*NPF4.6* in *Arabidopsis* was found to affect ABA import activity [39]. This suggested that a group of transporter genes up-regulated in *OsERF83-MYC^OX^* (Table 1, Figure 7a), including NPFs (nitrate and peptide transporters), might affect the drought tolerance of rice through the ABA-dependent pathway. On the other hand, recent reports have shown that lignin plays an important role in protection against water losses [40,41]. A group of lignin biosynthesis-associated genes up-regulated in *OsERF83-MYC^OX^* (Table 1, Figure 7d), including *OsLAC17* [42,43], *OsLAC10* [44], and *OsCAD8D* [45], might be involved in protection against water losses and affect drought tolerance. A number of abiotic stress-related genes containing *SENESCENCE-ASSOCIATED PROTEIN* (*OsSAP), OsLEA14/Wsi18, PCC13-62*, and heat shock protein were up-regulated in *OsERF83-MYC^OX^* (Table 1, Figure 7e). *OsSAP* is involved in drought tolerance by virtue of its antiapoptotic activity [46]. *OsLEA14/Wsi18* improves drought tolerance through higher proline and soluble sugar accumulation [47]. Desiccation-related protein (DRP)-encoding gene *PCC13-62* is involved in desiccation tolerance in *Linderniaceae* [48]. Heat shock proteins play a role in membrane stability and regulating antioxidant enzymes and are produced under various abiotic and biotic stresses [49,50]. It has been reported that *OsHSP23.7* enhances drought and salt tolerance in rice and overexpression transgenic plants show less membrane damage than NT plants [51]. It has also been shown that cytochrome P450 genes are involved in drought tolerance [52,53]. Many disease resistance-related genes, including *PATHOGENESIS-RELATED PROTEIN* (*PR*), *WALL-ASSOCIATED KINASE* (*WAK*) gene, *CELLULOSE SYNTHASE-LIKE PROTEIN E1* (*CslE1*), *LYSM RECEPTOR-LIKE KINASE* (*RLK*), and terpenoid-associated genes, were also up-regulated (Table 1). It has been reported that many stress-responsive genes are induced by both biotic and abiotic stress treatment. PRs correlate with drought tolerance [54,55]. Cell wall-associated genes containing *WAK*, *CslE1*, and lignin biosynthesis genes are involved in both abiotic and biotic stresses [56,57]. Accordingly, we speculated that *OsERF83* is involved in the core pathways in abiotic and biotic stress responses (Figure 8).

## 4. Materials and Methods

### 4.1. Plant Materials

To generate *OsERF83* overexpressing plants *(OsERF83^OX^*), the coding sequence of *OsERF83* (Os03g0860100, LOC_Os03g64260) was amplified from rice (*Oryza sativa* L. ssp. *Japonica* cv. Dongjin). The Japonica rice cultivar Dongjin (*Oryza sativa.* L.) was used as the non-transgenic control. The amplified *OsERF83* coding sequence was cloned into rice transformation vector *p700* carrying *GOS2* promoter for constitutive expression using the Gateway system (Invitrogen) (Figure S2). The final constructs were introduced into *Agrobacterium tumefaciens* LBA4404 by triparental mating and transformed into rice (*Oryza sativa* cv. Dongjin). Copy numbers were determined in T0 plants through Taq-Man PCR as described by [58].

For the generation of knock-out (*OsERF83^KO^*) mutants, we used the web-based tool CRISPR RGEN Tools (http://www.rgenome.net/, accessed on 18 May 2017). The method was performed as described previously [26].

### 4.2. Stress Treatments

To confirm the expression levels of the *OsERF83* gene under various abiotic stresses and phytohormone treatments, non-transgenic (NT) plants (*Oryza sativa* L. ssp. *Japonica* cv. Dongjin) were grown in soil for 2 weeks under standard greenhouse conditions (16 h light/8 h dark cycles at 28–30 °C). For stress treatments, the soil was moved from the roots of the seedlings, and drought stress was induced by air-drying the seedlings, while salinity stress and ABA treatment were imposed by incubating the seedlings in water containing 400 mM NaCl and 100 μM ABA, respectively at 28 °C. Low-temperature stress was induced by incubating the seedlings in water at 4 °C.

### 4.3. Subcellular Localization of OsERF83

To confirm the subcellular localization of OsERF83, we fused the coding regions of *OsERF83* without the stop codon to the *GFP.* The cassette was driven by a 35S promoter and inserted into the pHBT vector (GenBank accession number EF090408) using the In-fusion system (Clonetech, CA, USA). *35S::NF-YA7-mCherry* was used as the control for nuclear localization [23]. The final construct (*35S::OsERF83-GFP*) and the control vector (*35S::NF-YA7-mCherry*) were transfected into protoplasts (*Oryza sativa* cv. Dongjin) using a PEG-mediated protoplast transformation system [59]. The methods of rice protoplast preparation and transient gene expression were performed as described previously [60]. GFP and mCherry signals were observed 12 h after transfection using a confocal laser scanning microscope (Leica TCS SP8 STED, Wetzlar, Germany). Images were processed using Leica LAS AF Lite software. GFP was excited at 488 nm and the emitted light was detected between 512 and 560 nm. mCherry was excited at 587 nm and the emitted light was detected at 610 nm.

### 4.4. RNA Extraction, and Quantitative Real-Time PCR (qRT-PCR) Analysis

To investigate the spatial and temporal expression patterns of *OsERF83,* total RNA was isolated from the roots, stem, leaf, and flowers from different developmental stages of rice plants using a Hybrid-R RNA purification kit (GeneAll, Seoul, Korea) according to the manufacturer’s instructions. To measure the transcript levels of *OsERF83*, total RNA samples were extracted from the shoots and roots using a Hybrid-R RNA purification kit (GeneAll, Seoul, Korea) according to the manufacturer’s instructions. Complementary DNA (cDNA) was synthesized with Oligo-dT and random primers using RevertAid M-MuLV Reverse Transcriptase (Thermo Scientific, Massachusetts, USA). qRT-PCR was carried out using 2x qRT-PCR Pre-mix with 20x EvaGreen (SolGent, Seoul, Korea) and ROX dye (Promega, Madison, WI, USA). The amplification reactions were performed at 95 °C for 10 min, followed by 40 cycles of 95 °C for 30 s, 60 °C for 30 s, and 72 °C for 30 s in a 10 μL volume mix containing 0.5 μL EvaGreen Mix. The rice *Ubiquitin1* (AK121590, Os06g0681400) transcript was used as a normalization control, and two biological and three technical replicates were analyzed for all qRT-PCRs (Table S3).

### 4.5. Histochemical GUS Assay

The amplified promoter region of *OsERF83* was linked to the *GUS* reporter gene in a rice transformation vector using the Gateway system (Invitrogen, Carlsbad, CA, USA). The resulting plasmid was introduced into *A. tumefaciens* strain LBA 4404 by triparental mating and transformed into rice. To detect GUS staining, we used 5-day-old, 2-week-old, and 1-month-old seedlings of the *OsERF83::GUS* transgenic lines. The GUS staining solution contained 4 mM 5-bromo-4-chloro-3-indolyl-glucuronide cyclohexylamine salt (Gold Biotechnology, MO, USA), 0.5 mM K_3_Fe(CN)_6_, 0.5 mM C_6_FeK_4_N_6_, 0.1% Triton X-100, 10 mM EDTA, and 100 mM phosphate buffer. Seedlings were placed into GUS staining solution and then vacuum infiltrated for 30 min. Seedlings were then incubated at 37 °C in a dark chamber overnight. Chlorophyll was removed by washing with 70% (*v*/*v*) ethanol. We used Paraplast sections for longitudinals and cross-sections.

### 4.6. RNA-Sequencing Analysis

Three-week-old NT and *OsERF83* overexpression myc-tagged transgenic (*OsERF83-MYC^OX^*) plants were grown under standard greenhouse conditions (16 h light/8 h dark cycles at 28–30 °C). Total RNA was extracted from 3-week-old transgenic (*OsERF83-cMYC^OX^*) and non-transgenic (NT) roots and leaves using an RNeasy plant kit (Qiagen, Hilden, German). Two biological replicates were used for RNA-seq. Library construction and next-generation sequencing (NGS) were performed by the Macrogen facility (Seoul, Korea). To make expression profiles for *OsERF83-cMYC^OX^* plants, the fragment per transcript kilobase per million fragments mapped reads (FPKM) values for each transcript were normalized with the FPKM values. The expression level of each transcript was expressed as the fragment per transcript kilobase per million fragments mapped reads (FPKM) value, which was calculated based on the number of mapped reads.

### 4.7. Drought Stress Treatment and Measurement of Chlorophyll Fluorescence

We evaluated the drought tolerance of transgenic, knock-out, and non-transgenic (*Oryza sativa* L. ssp. *Japonica* cv. Dongjin) plants grown in a greenhouse at the vegetative stage. Transgenic, knock-out and non-transgenic seeds were germinated on Murashige and Skoog (MS) medium (Duchefa Biochemie, Haarlem, Netherlands) with 3% sucrose in the dark for 3 days at 28 °C and transferred into light conditions for 1 day. Thirty seedlings from each transgenic, knock-out mutant, and non-transgenic plant were transplanted into ten soil pots (4 × 4 × 6 cm, three plants per pot) within a container (59 × 38.5 × 15 cm) and grown for 5 weeks in greenhouse conditions (16 h light/8 h dark cycles at 28–30 °C). Drought stress was simultaneously imposed by withholding water and rewatering. Drought-induced symptoms were monitored by imaging plants at the indicated time points using an a5000 camera (Sony, Tokyo, Japan). Soil moisture was measured at the indicated time points using an SM 150 soil moisture sensor (Delta T Devices, Cambridge, United Kingdom).

To evaluate the phenotypes of transgenic, knock-out, and non-transgenic plants exposed to drought conditions, we measured the chlorophyll fluorescence (F_v_/F_m_) and the performance index (PI_total_). To measure F_v_/F_m_ and PI_total_, 4-week-old plants were transplanted into 15 cm diameter × 14 cm tall pots within another larger container (66 × 45.3 × 22.5 cm) and grown for 2 weeks. After each plant was subjected to drought stresses, F_v_/F_m_ and PI_total_ were measured using the Handy-PEA fluorimeter (Plant Efficiency Analyzer; Hansatech Instruments, King’s Lynn, Norfolk, United Kingdom) in dark conditions to ensure sufficient dark adaptation (at least 1 h). Nine leaves of each line were measured and calculated using the Handy PEA software (version 1.31) and analyzed according to the equations of the JIP test [61].

### 4.8. Statistical Analysis

All data are represented as mean ± standard deviation. Each data point was compared with the control separately to determine whether they were significantly different from each other, using Student’s *t*-test (* *p* < 0.05, ** *p* < 0.01). Data were analyzed by Microsoft Excel software.

### 4.9. Accession Numbers

Genes from this article can be found in the National Center for Biotechnology Information (http://www.ncbi.nlm.nih.gov/, accessed on 19 November 2020) with the following accession numbers: OsERF83 (Os03g0860100), OsNPF8.10 (Os01g0142800), OsNPF8.17 (Os10g0112500), OsLHT1 (Os08g0127100), OsMSL38 (Os11g0282700), OsMyb (Os01g0298400), OsTPS3 (Os02g0121700), OsCPS4 (Os04g0178300), OsLAC17 (Os10g0346300), OsLAC10 (Os02g0749700), OsCAD8D (Os09g0400400), OsSAP (Os09g0425900), PCC13-62 (Os04g0404400), and OsOPR4 (Os06g0215900).

## Figures and Tables

**Table 1:** List of genes up-regulated (>2-fold) in *OsERF83-MYC^OX^* and drought treatment in shoots and roots.

Descriptions	Gene ID	OX/NT Fc.	OX/NT Pval.	GCC Box
**Transporters**				
Similar to sugar transport protein 14	Os09g0322000	12.80	0.000	−1858, −1702, −902
OsLTP2.7	Os10g0504650	4.78	0.002	
OsNPF8.10	Os01g0142800	4.27	0.006	
OsATL11	Os02g0101000	3.95	0.003	
ABC transporter-like domain-containing protein	Os08g0564300	3.14	0.021	
NPF8.17	Os10g0112500	2.53	0.040	
OsACA1	Os03g0203700	2.34	0.044	−1439, −1052, −1049
NPF5.1	Os05g0336200	2.33	0.040	
OsLHT1	Os08g0127100	2.21	0.008	−1281
**Transcription factors**				
OsMSL38	Os11g0282700	25.08	0.000	−2906
Zinc finger, RING-type domain-containing protein	Os06g0534800	11.73	0.002	−2989, −2975
OsC3H32	Os04g0671800	10.06	0.000	−2976, −2918, −2879, −2876, −2835, −1283, −1144
Myb/SANT-like domain-containing protein	Os08g0496700	3.51	0.005	−1455, −1191, −900
OsC3H17, OsWD40–49	Os02g0677700	3.44	0.015	
OsbZIP89	Os12g0634500	3.27	0.028	−483
OsABI5, OsbZIP10	Os01g0859300	3.04	0.010	−1554
Zinc finger, RING/FYVE/PHD-type domain-containing protein	Os09g0504700	2.67	0.030	−1835, −1803, −1740
ONAC79	Os04g0437000	2.63	0.038	−2800, −506
OsERF86	Os07g0410700	2.62	0.029	−2589, −2495, −657
OsSPL1, OsDLN8	Os01g0292900	2.47	0.000	−2338, −2204, −1991
OsMyb	Os01g0298400	2.45	0.011	−1348, −1325, −1089, −889
OsbHLH025	Os01g0196300	2.41	0.046	−1581, −1578
Zinc finger, RING/FYVE/PHD-type domain-containing protein	Os07g0484300	2.16	0.045	−2923, −2920, −888
**Abiotic stress-related genes**				
OsDjA1	Os02g0656500	26.99	0.000	−2460, −2354, −2351
OsSAP, OsTET13	Os09g0425900	12.49	0.000	−908
OsTHIC	Os03g0679700	10.84	0.000	−1881, −1878, −1875
OsLEA14, wsi18	Os01g0705200	9.95	0.000	−2526
Similar to sorting nexin 1	Os01g0862300	6.86	0.000	−2778, −1217, −982, −979, −690, −544, −487, −481, −470, −359, −329, −298, −278, −188, −29
OsMPK14	Os05g0143500	6.85	0.013	
PCC13-62, OsEnS-64	Os04g0404400	5.60	0.000	−2980, −2977, −2974, −2223
OsHSP23.7	Os12g0569700	3.68	0.015	−1065, −1018, −988, −957, −444, −441, −379, −174
OsFAD2-3	Os07g0416900	3.43	0.005	−1476
Similar to class III peroxidase 136	Os12g0111800	3.29	0.001	
OsBBX28	Os09g0509700	2.65	0.009	−2975
OsNTP3	Os01g0846500	2.63	0.040	−1783, −1780, −1685, −1653
OsPEX11−5	Os06g0127000	2.49	0.019	−2371, −2368, −2054, −2051, −2048
Glycine-rich RNA-binding protein	Os04g0653700	2.37	0.032	−2957, −2901, −2895, −2892, −2307, −2684, −2680, −1677, −1610, −1607, −1604, −1577, −1478, −1475, −1442, −1415, −1322, −1319, −1316, −1313, −710, −678
OsSKIP	Os02g0759800	2.23	0.001	−2986, −2831, −1142, −1139, −1136, −990, −906, −840, −837, −800
OsRZFP34, OsRFP1	Os01g0719100	2.21	0.027	−1128, −1114, −346, −120
OsVPE3	Os02g0644000	2.20	0.000	−2797, −2751
OsASMT1	Os09g0344500	2.10	0.007	
**Cytochrome P450 family**				
Oscyp71Z4	Os10g0439800	5.79	0.001	−2642, −2625
Cytochrome P450 family protein	Os12g0582666	4.86	0.001	
CYP76M10	Os08g0507100	4.09	0.007	−1515, −1512, −1354, −1297, −1008, −572
CYP450-like Gene	Os08g0105700	4.09	0.000	−2870, −2337, −2065. −1006, −998, −995, −974
Similar to cytochrome P450 family protein, expressed	Os12g0640200	3.53	0.010	−1064, −1061, −1050, −989, −940, −240, −190, −187
OsCYP71AA2	Os01g0957800	3.50	0.022	−2440, −2437, −2434, −2410, −2224, −2029
Cytochrome P450 family protein	Os02g0184700	3.01	0.047	−1217
Cytochrome P450 family protein	Os03g0594100	2.54	0.022	−2178
CYP76M8	Os02g0569400	2.28	0.039	
CYP99A2	Os04g0180400	2.26	0.046	−2113, −2010, −1980, −1959
CYP76M2	Os08g0508000	2.15	0.012	−2928, −937
**Terpenoid-associated genes**				
OsTPS33	Os12g0491800	4.05	0.001	−608
OsTPS14	Os03g0428200	2.83	0.024	−1604
OsTPS3	Os02g0121700	2.80	0.027	
OsCPS4	Os04g0178300	2.68	0.000	−1983, −1933, −1926, −1897, −1825, −1797, −1794, −1786
OsTPS16	Os04g0179700	2.61	0.036	
**Disease resistance-related genes**				
OsCSN5A	Os04g0654700	25.76	0.000	
Similar to blight-associated protein p12	Os09g0472700	23.77	0.000	−2580, −1500, −1353, −1308, −1305, −1302, −1069, −1066, −1063, −995, −992, −927, −924, −130, −98, −37
OsLysM-RLK8	Os11g0549300	13.44	0.000	−2808, −2279, −2093, −2021, −1995, −1992, −1972
OsCslE1	Os09g0478100	10.69	0.000	−2802
eIF4Gtsv1	Os07g0555200	9.14	0.000	−1520
OsAGP29	Os01g0607100	7.42	0.000	−2606
OsWAK89b	Os09g0561450	6.55	0.005	
OsTHI7	Os06g0514100	5.48	0.004	−1693, −995
OsGLP4-1	Os04g0617900	5.22	0.000	−738
Similar to blight-associated protein p12 precursor	Os09g0472900	4.91	0.000	−2469, −2376, −178
Similar to receptor-like kinase Xa21-binding protein 3.	Os05g0112000	4.86	0.016	−2973
Similar to herbicide safener binding protein	Os11g0306400	4.67	0.042	
Leucine-rich repeat domain-containing protein	Os04g0122000	4.57	0.010	−1725
OsPR8	Os10g0416500	4.50	0.000	−2994, −2939, −871, −726
OsGSL3	Os01g0754200	4.45	0.018	−2773, −2770, −584, 581, −578, −575, 572, −569
OsEXO70B3	Os05g0473500	4.31	0.003	−2788, −2764, −2748, −508, −468, −465, −438, −413, −264, −159
Leucine-rich repeat, typical subtype domain-containing protein	Os11g0568800	3.83	0.009	−2429, −2426, −1239, −1210, −1185, −1130
Leucine-rich repeat, N-terminal domain-containing protein	Os12g0211500	3.80	0.017	
Similar to pathogen-related protein	Os01g0248300	3.80	0.000	−2698, −2680, −2644, −2637, −1700, −1620, −1560, −1557, −1534, −1317
OsDXS3	Os07g0190000	3.28	0.000	
Multi-antimicrobial extrusion protein MatE family protein	Os09g0524300	3.27	0.026	
OsLOX9	Os08g0508800	3.20	0.000	−1626
OsWAKL21.2	Os12g0595800	2.93	0.016	−959, −842, −839, −836, −833, −308, −305, −302, −299, −296, −183, −167, −164, −161, −92
Similar to resistance protein candidate (fragment)	Os03g0258000	2.84	0.031	−577, −478, −391
IAI2	Os01g0132000	2.78	0.000	−1174, −1167
OsTHI9	Os06g0514800	2.74	0.025	−1718, −1715, −1697
Leucine-rich repeat, N-terminal domain-containing protein	Os01g0891700	2.70	0.031	
OsPR4d	Os11g0591800	2.26	0.001	−2134, −993
Allergen V5/Tpx-1 related family protein	Os07g0126401	2.24	0.014	
Allergen V5/Tpx-1 related family protein	Os07g0125600	2.23	0.015	−553, −386, −336, −333, −215
OsPR1b	Os07g0125000	2.22	0.015	
OsPR1-73	Os07g0127600	2.15	0.018	
Similar to thaumatin-like protein	Os12g0630500	2.15	0.000	−2453
PR5	Os03g0663500	2.12	0.006	
OsGLN1	Os01g0946700	2.06	0.000	−2549, −1263, −760, −669, −616, −457, −374, −323
OsAOS2	Os03g0225900	2.01	0.007	−2843, −2202, −349
**Hormone-associated genes**				
OsLUGL, OsWD40-17	Os01g0607400	11.97	0.001	−2980, −2906, −2903, −2900, −2897, −2836, −2833
OsARF12	Os04g0671900	10.33	0.000	
OsJAZ8	Os09g0401300	6.01	0.000	−2541, −2538
OsGA20ox4	Os05g0421900	4.85	0.015	
OsARF13, OsARF12	Os04g0690600	2.74	0.030	
OsOPR4	Os06g0215900	2.46	0.000	−2863, −1540, −1481, −1478, −1459, −1431, −1428, −1279, −1276, −1238, −1206, −1133, −1129, −1126, −1070, −1040, −1016, −1013
**Lignin biosynthesis-associated genes**				
OsUGT707A5	Os07g0503300	4.53	0.000	−1544, −1507, −1470
OsUGT93B	Os04g0556400	3.99	0.003	−2364
UDP-glucuronosyl/UDP-glucosyltransferase family protein	Os03g0804900	3.23	0.027	−2977, −805, −777, −774, −740, −737, −690, −473, −470, −407, −404, −376
OsLAC17	Os10g0346300	3.02	0.010	−1525, −1481, −1468, −151
OsLAC10	Os02g0749700	2.95	0.026	−1963, −1907
OsCAD8D	Os09g0400400	2.94	0.000	−2137
**F-box protein**				
OsFbox331	Os07g0118900	8.11	0.013	
OsFbox228	Os04g0571300	4.07	0.003	−1080
OsFbox552	Os10g0396400	3.34	0.036	

## Data Availability

Not applicable.

## Supplementary Materials

The following are available online at https://www.mdpi.com/article/10.3390/ijms22147656/s1.
