# Supplementary material for: Overexpression of OsERF83, a Vascular Tissue-Specific Transcription Factor Gene, Confers Drought Tolerance in Rice
Source: Int J Mol Sci. 2021 Jul 17;22(14):7656. doi: 10.3390/ijms22147656 (PMC8304134; doi:10.3390/ijms22147656)
Supplement: Supplementary file 1 [file ijms-22-07656-s001.zip › ijms-1284448-final Supplementary Materials.pdf]

Supplementary Materials

Overexpression of OsERF83, a vascular tissue-specific transcription factor gene, confers drought tolerance in rice

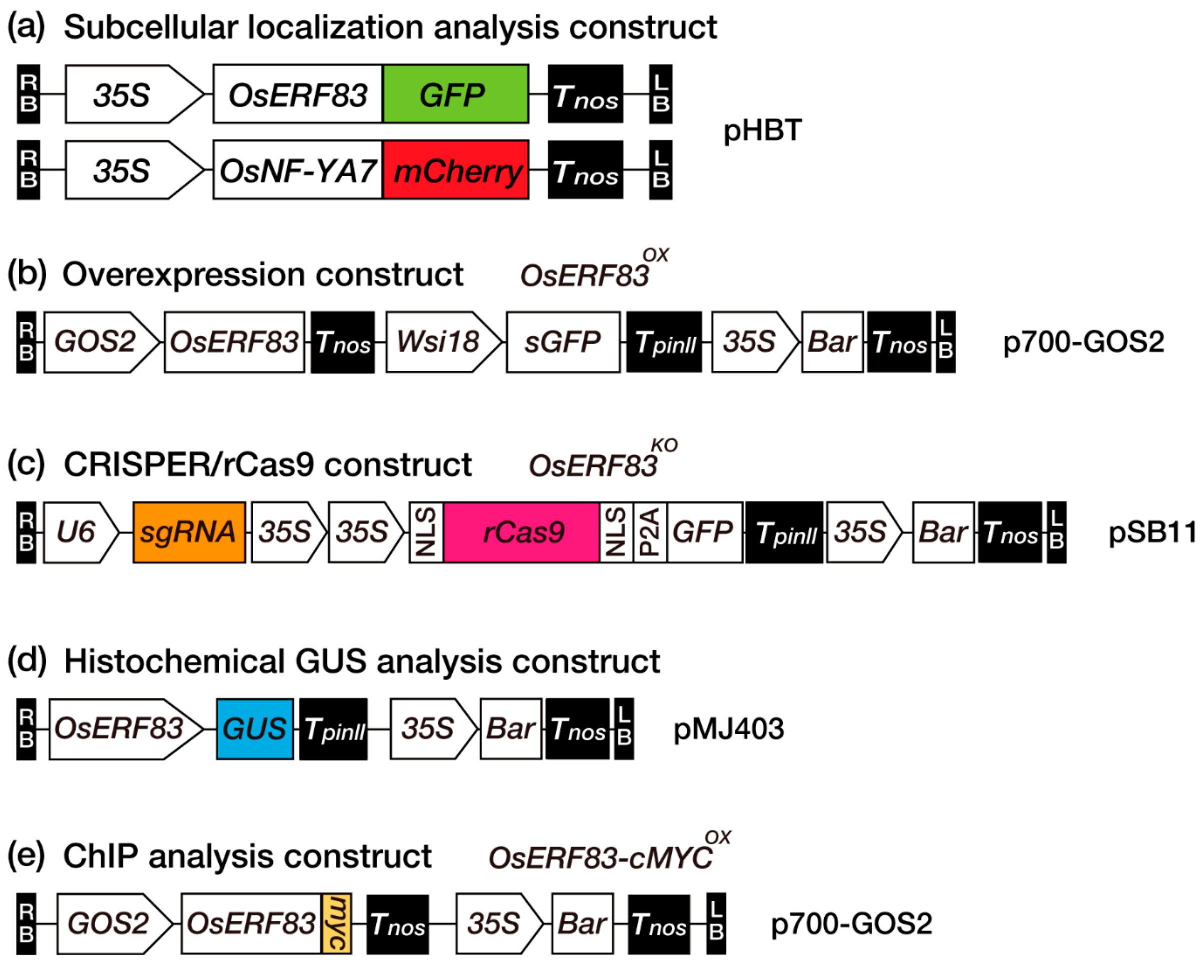

Figure S1. Schematic diagram of the vector constructs used in this study. *GOS2*, promoter of rice *eukaryotic translation initiation factor 1-like gene* (Os07g0529800); *Tnos*, the 3' region of nopaline synthase gene; *TpinII*, the 3' region of the potato (Solanum tuberosum) proteinase inhibitor II gene; *35S*, 35S promoter of Cauliflower mosaic virus; *Bar*, the bacterial phosphinothricin acetyltransferase gene; *Wsi18*, promoter of *a stress-inducible gene*; LB, left border; RB, right border; T, terminator.

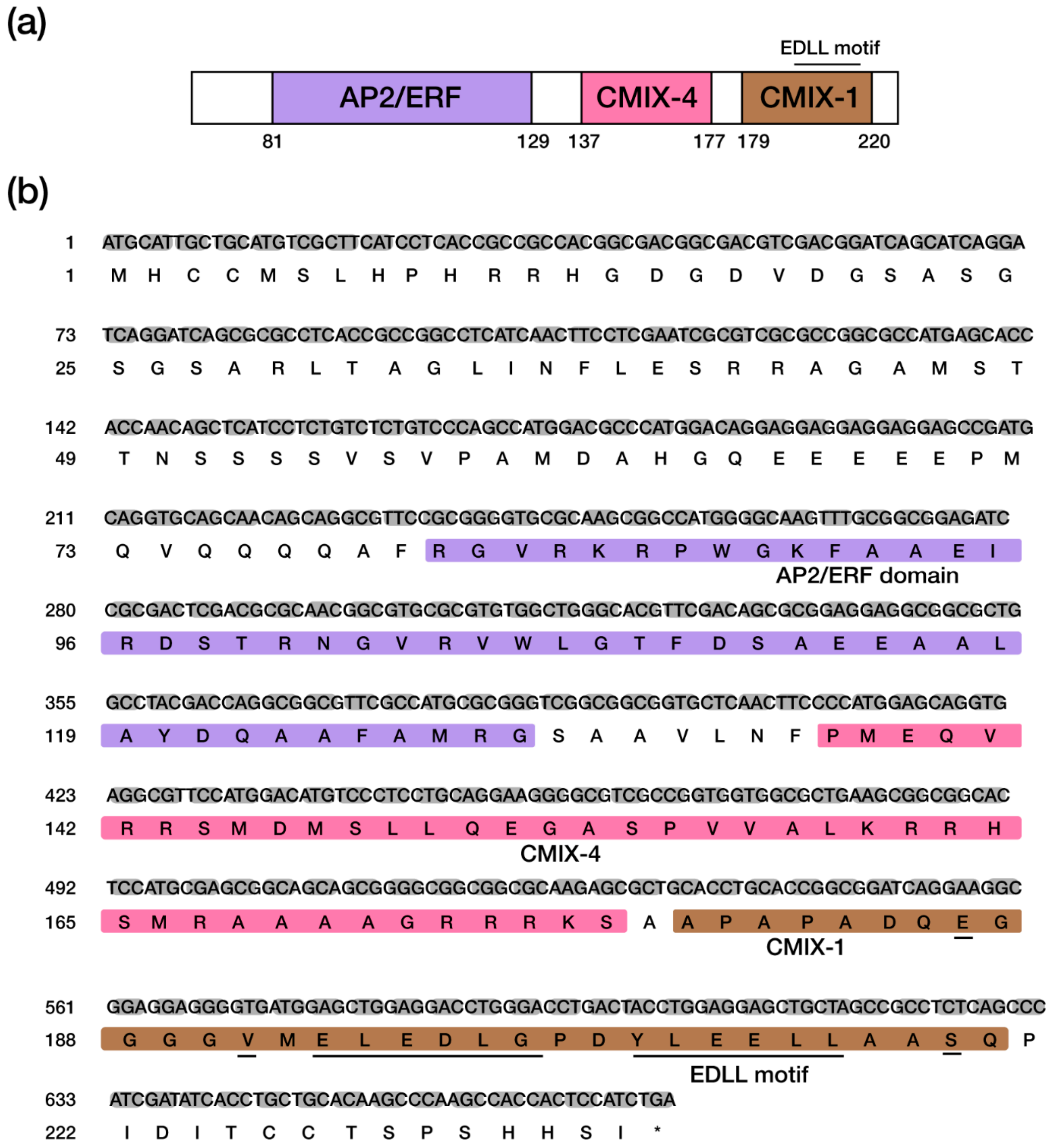

Figure S2. The structure of domains and motifs of *OsERF83*. (A) A schematic representation of domains and motif based on (Nakano *et al.*, 2006) is shown. The box indicates the CDS of *OsERF83.* The functional domains are colored and the motif is represented as a bar. (B) The amino acid and nucleotide sequences of the *OsERF83* are aligned. Gray small circle-shaped box indicates the set of the codon. The purple box indicates AP2/ERF domain, Pink and

brown boxes represent CMIX-4, -1 domain, respectively. EDLL motif is indicated in CMIX-1 domain at the end of C-terminal.

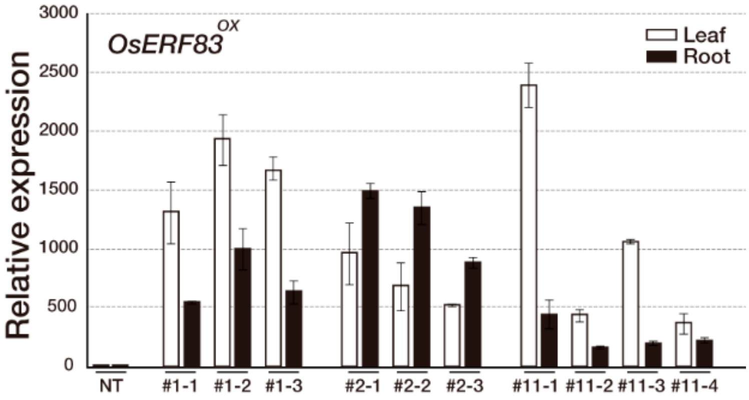

Figure S3. Relative expression levels of *OsERF83* in non-transgenic (NT) and three independent *OsERF83* overexpressed (*OsERF83*^{OX}) transgenic rice plants. Total RNAs extracted from leaves and roots of each sister line. *OsUbi1* was used as the internal control for normalization. Data represent mean value ± *SD* (n=3).

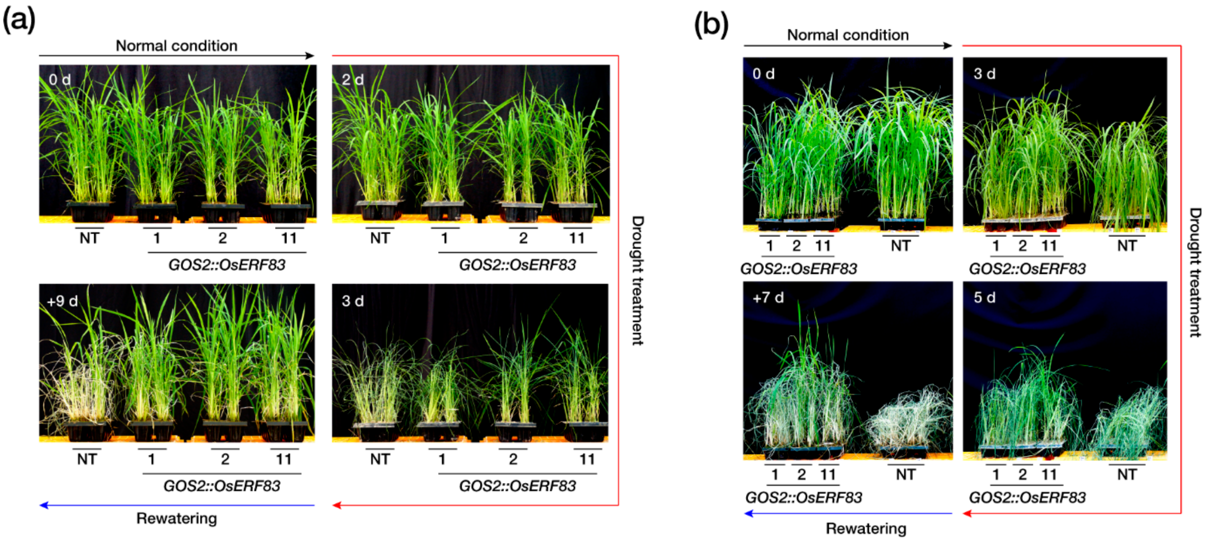

Figure S4. Phenotypes of the T3 (left panel), and T4 (right panel) generations of *OsERF83*^{OX} transgenic rice and NT plants under drought treatments.

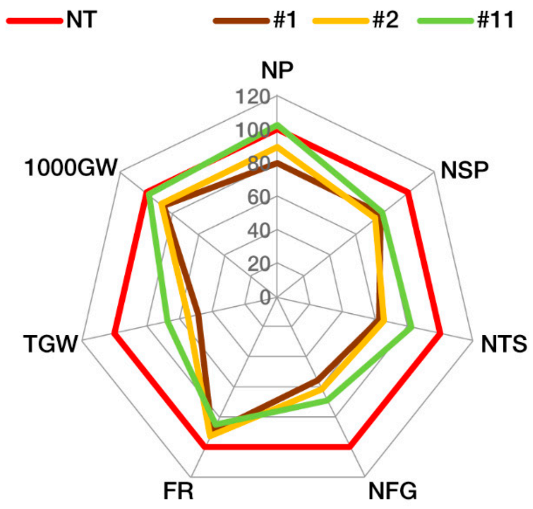

Figure S5. Agronomic traits of three independent T4 homozygous *OsERF83*^{OX} transgenic rice (lines 1, 2, 11) compared with NT in the paddy field (2020). Each data point represents the percentage of the mean values (n=18) listed in Table S2. Mean values from NT plants were assigned a reference value of 100%. NP, number of panicles per hill; NSP, number of spikelets per panicle; NTS, number of total spikelets; NFG, number of filled grains; FR, filling rate; TGW. Total grain weight; 1000 GW, 1000 grain weight.

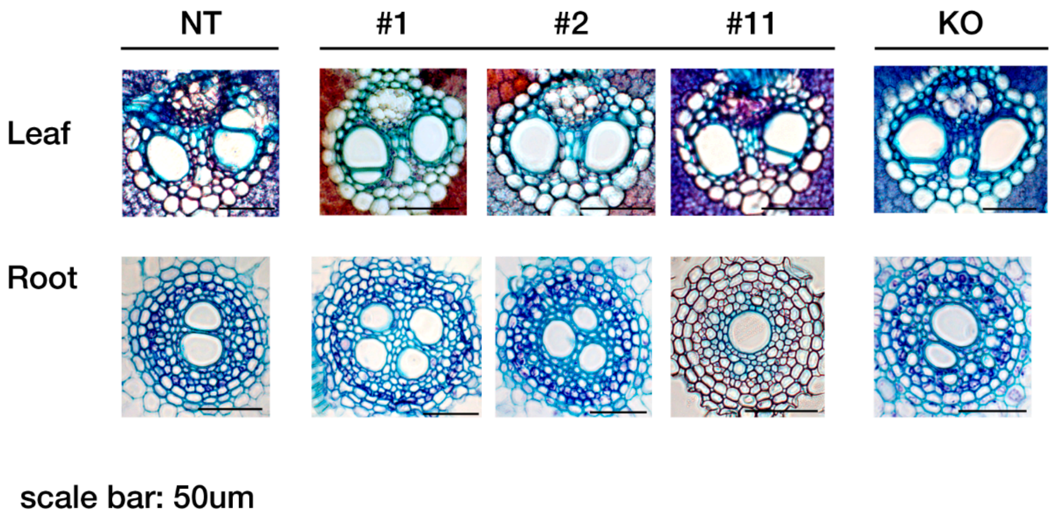

Figure S6. Cross-section images showing 2-month old mature leaves (upper panels) and roots (bottom panels) of NT, *OsERF83*^{OX} transgenic rice, and knock-out (*OsERF83*^{KO}) mutants. Scale bar represents 50μm

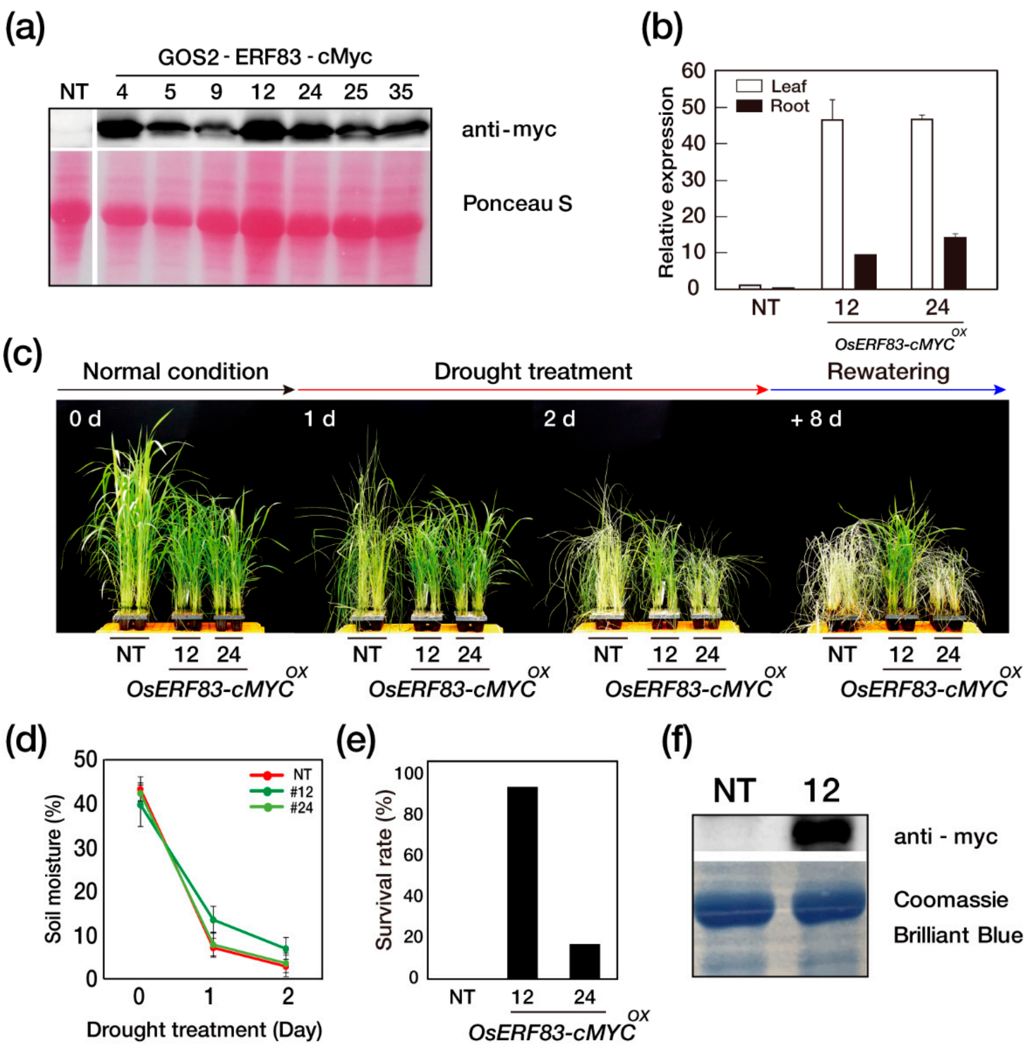

Figure S7. The protein expression of *OsERF83* overexpression myc tagging transgenic (*OsERF83-MYC^{OX})* and the phenotype under drought treatment. (a) Western blot analysis of *OsERF83-MYC^{OX}* (lines 4, 5, 9, 12, 24, 25, 35) plants leaves with anti-myc antibody. The upper panel showed OsERF83-myc recombinant proteins in blot and the lower panel showed Ponceau S staining as a loading control of each line. (b) Relative expression levels of *OsERF83-MYC^{OX}* (lines 12, 24). *OsUbi1* (*Ubiquitin1*; Os06g0681400) was used as the internal control for normalization. Data represent mean value + *SD (n=3)* (c) Drought tolerance phenotypes of *OsERF83-MYC^{OX}* (lines 12, 24) transgenic plants. All plants were grown in soil 5 weeks under a well-watered condition and exposed to drought stress for 2 days, followed by re-watering for 8 days in the greenhouse. (d) Measurement of soil moisture

contents (%). Data represent mean value ± SD of 15 measurements performed at different pots. (e) The survival rate of *OsERF83-MYC*^{OX} (lines 12, 24) transgenic plants scored 8 days after re-watering. (f) Western blot analysis of *OsERF83-MYC*^{OX} (lines 12, 24) plants from (c) leaves with anti-myc antibody.

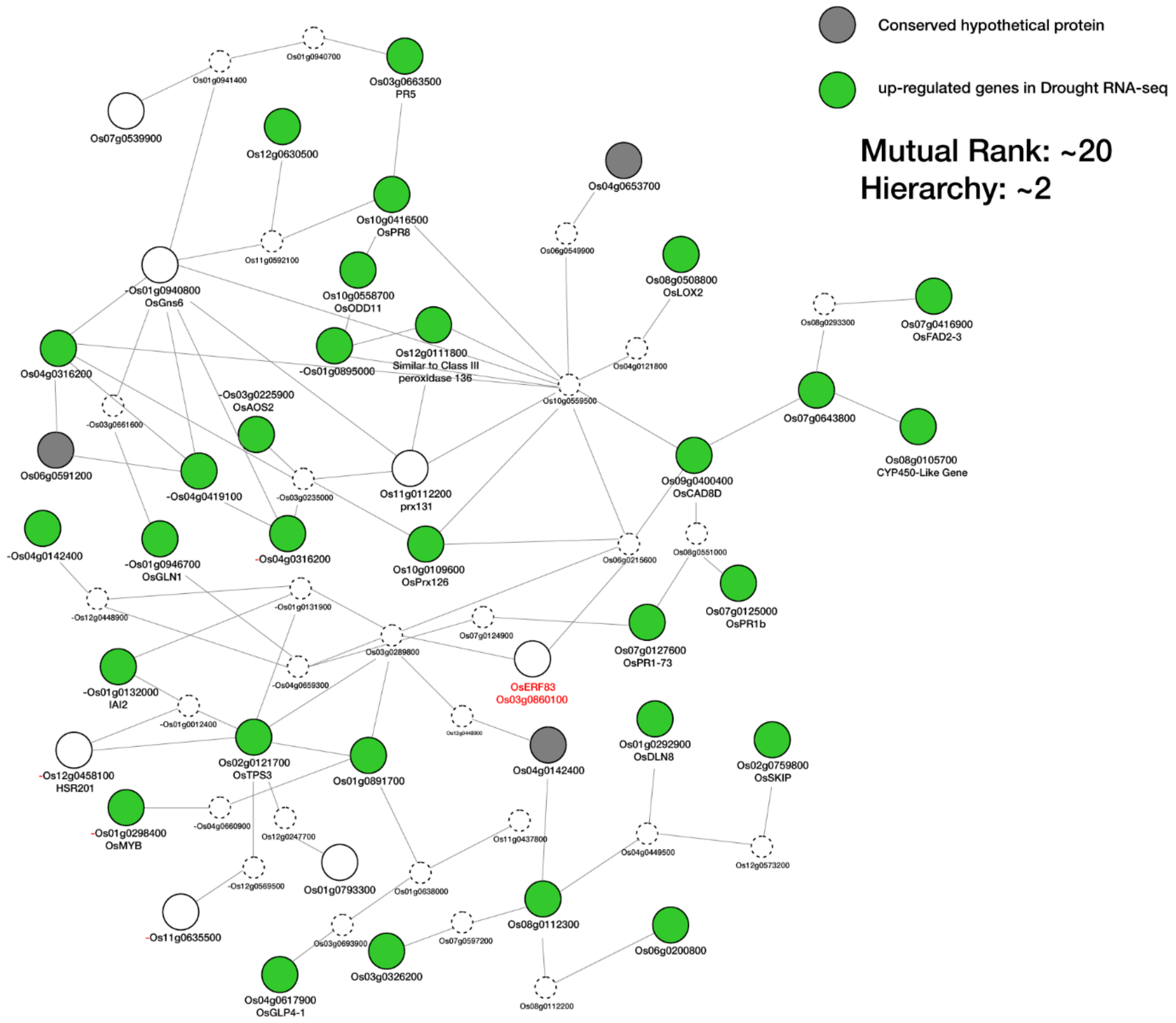

Figure S8. *OsERF83* transcriptional co-regulatory network. Co-regulatory genes were selected from differentially expressed genes (DEGs) in *OsERF83-MYC*^{OX}.

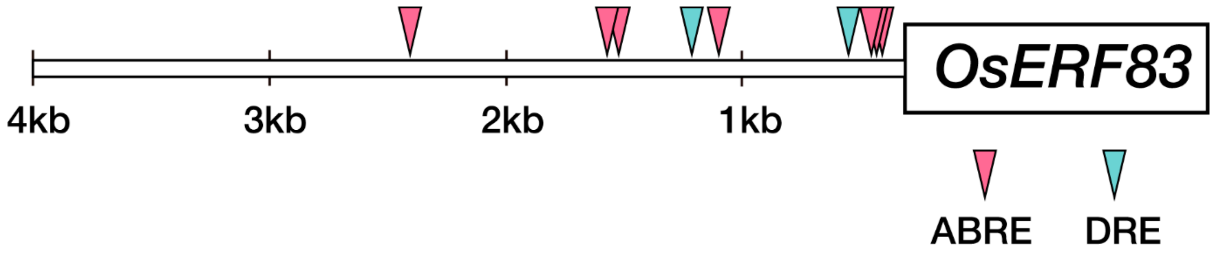

Figure S9. Cis-elements of a promoter in *OsERF83.* A dehydration-responsive element (DRE; TACCGACAT) and ABA-responsive element (ABRE; ACGTGG/TC) cis-elements were represented.

Table S1. Agronomic traits of *OsERF83* overexpression transgenic rice plants grown under normal conditions.

Genotype	Plant height (cm)	Culm length (cm)	Panicle length (cm)	No. of panicle /hill	No. of spikelet/ panicle	No. of total spikelet/ hill	Number of filled grains (NFG)	Filling rate (%)	Total grain weight (g)	1000 grain weight (g)
NT	98.86	78.71	20.14	17.55	104.52	1783.90	1507.00	84.95	35.75	23.76
OX-#1	75.50	59.67	15.7	14.00	82.91	1116.50	841.55	76.56	17.35	20.65
% △	-23.6	-24.19	-22.05	-20.23	-20.68	-37.41	-44.16	-9.88	-51.49	-13.10
p-value	0.000**	0.000**	0.001**	0.006**	0.011*	0.000**	0.000**	0.000**	0.000**	0.000**
OX-#2	87.63	71.69	15.94	15.67	78.33	1168.50	928.83	78.71	19.57	21.09
% △	-11.4	-8.92	-20.86	-10.73	-25.06	-34.50	-38.37	-7.34	-45.25	-11.24
p-value	0.000**	0.006**	0.000**	0.242	0.000**	0.000**	0.000**	0.000**	0.000**	0.000**
OX-#11	81.50	63.06	18.44	18.00	84.08	1468.74	1040.74	72.36	24.07	23.31
% △	-17.6	-19.88	-8.45	2.56	-19.56	-17.67	-30.94	-14.82	-32.68	-1.89
p-value	0.000**	0.000**	0.020*	0.743	0.014*	0.029*	0.000**	0.000**	0.000**	0.344

Table S2. List of other genes up-regulated (> 2 fold) in *OsERF83-MYC*^{OX} compared with NT, following drought treatment in shoots and roots.

Descriptions	Gene ID	OX/NT fc.	OX/NT pval.	GCC box
**Others**				
OsSNDP6	Os05g0545000	93.56	0.000	-2547
OsCDC48, PSD128	Os03g0151800	23.44	0.000	
OsASL1	Os03g0305500	8.59	0.001	-2950, -1607, -1491, -1488, -1365, -1362, -1302, -1158, -941, -328, -317, -234, -231, -214, -181, -178, -175, -27, -24
OsNABP	Os06g0215200	7.42	0.018	
OsSCP65, CBP1	Os12g0257000	6.16	0.034	-1760, -1493, -1421, -1306, -1260, -1176, -1086, -1083, -1039, -1036, -1002, -975
OsRab5B1	Os03g0666500	6.12	0.000	
OsVLN4	Os04g0604000	5.38	0.000	-1293, -843, -840
OsalphaCA3	Os08g0423500	4.72	0.025	-506, -472, -464, -434
OsPP2C14, OsPP24	Os02g0471500	4.42	0.028	-2409, -156
Glua1, OsEnS-16	Os01g0762500	4.26	0.042	
OSK28	Os07g0625400	3.98	0.001	-2892, -2823, -470, -467, -364, -129
OsRFP	Os03g0326300	3.82	0.050	
OsEnS-40	Os02g0586900	3.74	0.003	
OsTHI3, OsTHION3	Os06g0513050	3.59	0.006	
OsEnS-119	Os08g0286500	3.31	0.013	
OsPGK1	Os01g0800266	3.29	0.037	-201, -192
OsPUP2	Os09g0467300	3.17	0.040	
OsGSTF5	Os01g0369700	3.15	0.001	
OsTBL33	Os12g0516800	3.14	0.007	-1155, -1017, -1013
Osppc2a, OSPPC	Os08g0366000	3.09	0.017	
Tryptophan synthase	Os08g0135900	3.07	0.005	
OsCatB	Os05g0310500	2.98	0.000	-2294
Similar to Stearoyl-acyl carrier protein desaturse	Os03g0423300	2.91	0.031	
Similar to Oxidoreductase, 2OG-Fe oxygenase family protein, expressed	Os10g0558750	2.64	0.000	
OsPAP27a, OsNPP6	Os09g0506000	2.60	0.046	-2870, -2830, -2827, -1219, -1156, -1153, -1138, -1090, -1087, -1084, -1081
OsISC3	Os09g0270900	2.57	0.005	-2951, -2926, -2867, -2864, -2861
Similar to ACX4 (ACYL-COA OXIDASE 4); acyl-CoA oxidase/oxidoreductase	Os06g0346300	2.56	0.026	-280, -277, -213, -179, -161, -108, -90
OsSTA16	Os01g0548000	2.49	0.035	-2323
OsRePRP2.1	Os07g0418700	2.49	0.001	x
Arf GTPase activating protein family protein	Os07g0563800	2.48	0.000	-552, -530, -358, -334, -327, -320, -295

OsPP2A-B''	Os10g0476600	2.46	0.011	-2887, -2836, -2802, -2781, -856, -114, -111
OsTHI5, OsTHION4	Os06g0513862	2.33	0.043	-1718, -1715, -1697, -1002, -999
Zinc finger, RING/FYVE/PHD-type domain containing protein	Os06g0717600	2.11	0.006	-2908
OsCBSCBS3	Os04g0382300	2.07	0.000	-2575, -2572, -2390
OsaHMT4	Os12g0607000	2.04	0.005	
OsODD11, OsM3H	Os10g0558700	2.01	0.000	

Table S3. List of primers used in this study.

Gene	Primer Sequence		
	**Purpose**	**Forward**	**Reverse**
OsERF83 (Os03g0860100)	qRT-PCR	5'-GACGGATCAGCATCAGGATCA-3'	5'-TCCATGGCTGGGACAGAGAC-3'
Ubi (Os06g0681400)	qRT-PCR	5'-GCCAAGATCCAGGACAAGGA-3'	5'-GCCATCCTCCAGCTGCTT-3'
OsNPF8.10 (Os01g0142800)	qRT-PCR	5'-TGCTGCCTGTTCAGTTTCTC -3'	5'-CCAAGCATTCCGTCCCTGAT-3'
OsNPF8.17 (Os10g0112500)	qRT-PCR	5'-GGGATGATCACGCTCACAGT-3'	5'-AGGTAGAGCCCCAGGAACAC-3'
OsLHT1 (Os08g0127100)	qRT-PCR	5'-AAGAAGTTCCACGACGTGCT-3'	5'-GTTGAAGTTTGGGAGCTGCG-3'
OsMSL38 (Os11g0282700)	qRT-PCR	5'-CCTAAAACTAAGGTATCGAAGATGC-3'	5'-ATTCATAGGCCATAACAGTGACC-3'
Myb/SANT-like domain domain containing protein. (Os08g0496700)	qRT-PCR	5'-GGGCTAAATTCATTAACGTCCCC-3'	5'-TCAACATCACCAGGACTGCC-3'
OsMYB (Os01g0298400)	qRT-PCR	5'-ACGAACCACCACCTGATGAC-3'	5'-AACTGATCCCAACGCTCGTG-3'
OsTPS3 (Os02g0121700)	qRT-PCR	5'-ATGGCGAATTCAGGCTCGAT-3'	5'-TGCAAGATTTGGCTCGAGGT-3'
OsCPS4 (Os04g0178300)	qRT-PCR	5'-GGGTTCTACAATTAATGTTCCGGT-3'	5'-ACAGACAGCATGCACTGTCA-3'
OsLAC17 (Os10g0346300)	qRT-PCR	5'-ACCCAGGGGCATGGTTAATG-3'	5'-TCATGGCCTTCCGTCCTAGA-3'
OsLAC10 (Os02g0749700)	qRT-PCR	5'-CAGCTTACGGCACTAGTTAGC-3'	5'-CCACGCCTGCTAGTAACCAA-3'
CAD8D (Os09g0400400)	qRT-PCR	5'-GCTTAGCAAGGAGTGGTCGAT-3'	5'-TGATGATGTGCAGGTCGGT-3'
OsSAP (Os09g0425900)	qRT-PCR	5'-GGGATCGGACCCAGTTCTTC-3'	5'-GACTGGCAGTTGTAGCACAG-3'
Similar to Sorting nexin 1 (Os01g0862300)	qRT-PCR	5'-AAAAATAATTCGGGCAGGATCTC-3'	5'-GGCTCAGTTGAACCTCCCAA-3'
PCC13-62 (Os04g0404400)	qRT-PCR	5'-TGCTACCAAGAAGTCGGCCA-3'	5'-GCTGTTCTCGTAGGGGTTGAA-3'
OsOPR4 (Os06g0215900)	qRT-PCR	5'-CCTACGGGAGGCACTTCTTG-3'	5'-CATAAGTAGCAGCGCCCTCG-3'
OsERF83 (Os03g0860100)	CDS	5'-GCATTGCTGCATGTCGCTTC-3'	5'-GCTGTTGGTGGTGCTCATGG-3'

Reference

Nakano T, Suzuki K, Fujimura T, Shinshi H. 2006. Genome-Wide Analysis of the ERF Gene Family in Arabidopsis and Rice. Plant Physiology **140**, 411-432.
